# Diagnostic profiles among suicide decedents with and without borderline personality disorder

**DOI:** 10.1017/S0033291724002034

**Published:** 2024-11

**Authors:** Erin A. Kaufman, Hilary Coon, Andrey A. Shabalin, Eric T. Monson, Danli Chen, Michael J. Staley, Brooks R. Keeshin, Anna R. Docherty, Amanda V. Bakian, Emily DiBlasi

**Affiliations:** 1 Department of Psychiatry & Huntsman Mental Health Institute, Spencer Fox Eccles School of Medicine, University of Utah, Salt Lake City, UT, USA; 2 Office of the Medical Examiner, Utah Department of Health and Human Services, Salt Lake City, UT, USA; 3 Safe and Healthy Families, Primary Children's Hospital, Intermountain Healthcare, Salt Lake City, UT, USA; 4 Department of Pediatrics, University of Utah, Salt Lake City, UT, USA; 5 Clinical and Translational Research Institute, University of Utah Health, Salt Lake City, UT, USA

**Keywords:** borderline personality disorder, self-harm, suicidal behavior, suicide attempt, suicide

## Abstract

**Background:**

Borderline personality disorder (BPD) is a debilitating condition characterized by pervasive instability across multiple major domains of functioning. The majority of persons with BPD engage in self-injury and up to 10% die by suicide – rendering persons with this condition at exceptionally elevated risk of comorbidity and premature mortality. Better characterization of clinical risk factors among persons with BPD who die by suicide is urgently needed.

**Methods:**

We examined patterns of medical and psychiatric diagnoses (1580 to 1700 Phecodes) among persons with BPD who died by suicide (*n* = 379) via a large suicide death data resource and biobank. In phenotype-based phenome-wide association tests, we compared these individuals to three other groups: (1) persons who died by suicide without a history of BPD (*n* = 9468), (2) persons still living with a history of BPD diagnosis (*n* = 280), and (3) persons who died by suicide with a different personality disorder (other PD *n* = 589).

**Results:**

Multivariable logistic regression models revealed that persons with BPD who died by suicide were more likely to present with co-occurring psychiatric diagnoses, and have a documented history of self-harm in the medical system prior to death, relative to suicides without BPD. Posttraumatic stress disorder was more elevated among those with BPD who died by suicide relative to the other PD group.

**Conclusions:**

We found significant differences among persons with BPD who died by suicide and all other comparison groups. Such differences may be clinically informative for identifying high-risk subtypes and providing targeted intervention approaches.

## Introduction

Borderline personality disorder (BPD) is a debilitating psychiatric condition characterized by pervasive instability across multiple core domains of functioning. Although a heterogeneous disorder, those affected by BPD typically experience intense and frequent emotion dysregulation, interpersonal strife, and engage in self-damaging behaviors (American Psychiatric Association [APA], 2022). As many as 10% of persons with BPD are estimated to die by suicide (Paris, 2019). Persons with BPD frequently utilize mental health resources, emergency departments, and primary healthcare systems (Pascual et al., 2007; Sansone, Farukhi, & Wiederman, 2011), and thus, clinical settings offer a unique opportunity to prevent premature mortality among this population.

Many risk and vulnerability factors thought to shape the emergence and maintenance of BPD are also linked to the etiology of self-inflicted injury, and suicide more broadly (e.g. emotion dysregulation, interpersonal trauma, isolation, and loss, behavioral impulsivity; Derbidge & Beauchaine, 2014; Kaufman, Crowell, & Lenzenweger, 2017). This co-occurrence helps to explain why suicide risk is elevated among persons with BPD compared to many other clinical groups and compared to the general population (Kaufman et al., 2017). At the same time, the majority of persons who die by suicide do not have a BPD diagnosis, and the majority of those with a BPD diagnosis do not die by suicide. Given that suicide is a low base-rate outcome even among high-risk groups (Centers for Disease Control and Prevention, 2023), and key predictors do not typically demonstrate sensitivity or account for substantial variance in suicide in isolation (Franklin et al., 2017), identifying risk *profiles* among vulnerable groups may be particularly beneficial. Recent work by Xiao et al. supports this multifaceted approach to suicide prevention. The authors identified five distinct suicide profiles (latent classes) in an analysis of data (death investigator information gathered from witnesses, family, friends, and other informants) on over 300 000 suicide decedents from the National Violent Death Reporting System 2003–2020. This work highlights the complexity of suicide and the importance of suicide prevention strategies that address the specific and often differing needs of those who die by suicide.

Specific to BPD, Wedig et al. (2012) assessed baseline and time-varying predictors of suicide attempts among patients with BPD over 16 years of prospective follow-up. They identified several risks as bivariate predictors of suicide attempts, many of which were co-occurring mental health conditions and previous exposure to self-injury. It can be challenging to identify persons with BPD who are at particularly high risk of death by suicide, as, by definition, many of the risk factors for self-injury are defining criteria of BPD. It therefore may be helpful to characterize how persons with BPD who die by suicide differ both from living individuals with BPD and those who die by suicide without a history of this condition.

Although it is challenging to design prospective studies that will sample persons who do and do not die by suicide, alternative designs using retrospective medical records often contain important information that can speak to individuals' functioning prior to death. The present study utilizes a population-based data resource and biobank available through the Utah Suicide Mortality Risk Study (USMRS) to characterize patterns of medical and mental diagnoses among persons with a BPD diagnosis who died by suicide (SUI + BPD). To better understand the factors that are associated with suicide death among this already high-risk group, we compare our SUI + BPD group to (1) persons who died by suicide without a history of BPD (SUI – BPD), (2) persons with BPD who did not die by suicide (CTRL + BPD), and (3) individuals who died by suicide with a different personality disorder diagnosis (SUI + PD – BPD) using a phenotype-based phenome-wide association study framework. Identifying high-risk profiles or subtypes of individuals who are particularly vulnerable to suicide may help us more effectively target prevention and treatment efforts that are typically limited in availability.

## Methods

### Samples

Suicide mortality is particularly high in the state of Utah (Utah Department of Health and Human Services, Indicator-Based Information System (IBIS) for Public Health, 2023). A centralized Office of the Medical Examiner (OME) makes a determination of suicide following a detailed investigation of the scene and circumstances of death, and determination of medical conditions by autopsy, interviews with survivors, and toxicology reports. Through a 25-year collaboration with the OME, the USMRS has built a large population-based data resource that facilitates unique opportunities to identify critical gaps in our understanding of suicide and enhance ongoing suicide prevention work. Annual approvals from institutional review boards at the University of Utah, Intermountain Healthcare, and the Utah Department of Health and Human Services allow the linkage of Protected Health Information (PHI) from suicide deaths to data within the Utah Population Database (UPDB). The UPDB is a secure state-wide database that includes demographics and two decades of electronic health records (EHR) data (all inpatient encounters, emergency department, and ambulatory care encounters, and outpatient encounters from the two largest clinical data providers in the state, University of Utah Healthcare and Intermountain Healthcare), with coverage of ~85% of the state's population. PHI is stripped before data are provided to the research team and all samples are de-identified. Currently, the USMRS includes records from 10 500 Utah suicides with linked demographic and EHR data. In addition to the suicide death data, demographic and EHR data are also available from 99 990 Utah controls (population-based; matching 1:10 on age/sex to suicide deaths using at-risk sampling). This study assessed suicide death and control data from 1996 through 2022.

### Diagnostic EHR data

Phenotypes for suicide deaths and controls were based on International Classification of Diseases (ICD 9 and 10) diagnostic codes. For suicide deaths, ICD codes within 2 weeks of death were removed to ensure that codes from the death event were not included in the phenotyping. Only individuals who were 18 and older were included in this study. The presence of at least one BPD ICD code (ICD 9 301.83; ICD10 F60.3) was used to define individuals with a BPD diagnosis. To broadly assess common medical conditions, diagnostic data were aggregated to PheWAS trait codes (hereby ‘phecode’). Phecodes are curated groupings of ICD codes designed to capture clinically relevant traits or conditions and are commonly used in phenotyping applications from the EHR (Bastarache, 2021). We used the PheWAS R package (Carroll, Bastarache, & Denny, 2014) for suicides and controls separately. Phecodes represent one validated way to define phenotypes for research using EHR data (Bastarache, 2021). ICD 9 Phecode Map 1.2 was used to aggregate all ICD 9 codes and ICD 10 CM Phecode Map 1.2 beta was used to aggregate all ICD 10 codes (Denny et al., 2013). Phecode maps, phecode definitions, and additional information can be found at https://phewascatalog.org/phecodes. Individuals were classified as positive for a specific phecode trait if their medical records contained at least one corresponding ICD code within that phecode's defined set, ensuring a comprehensive phenotypic categorization based on established clinical coding standards. We defined the SUI + PD−BPD group based on the presence of at least one ICD code in the personality disorder phecode domains (‘Personality disorders’, ‘Schizoid personality disorder’, or ‘Antisocial/borderline personality disorder’), only including individuals *without* an ICD BPD diagnosis. Other personality disorders in ICD 9 and 10 include paranoid, schizoid, schizotypal, antisocial, borderline, histrionic, narcissistic, avoidant, dependent, and obsessive-compulsive PDs, as well as other specific or unspecified personality disorders (see online Supplementary Table S1). Comparisons of age and sex between the groups (SUI + BPD *v.* SUI−BPD; SUI + BPD *v.* CTRL + BPD; SUI + BPD *v.* SUI + PD−BPD) were done using *t* tests with Benjamini–Hochberg (BH) adjustment to correct for multiple testing. The mean time to death from the first diagnosis year of BPD and the year of suicide death was also calculated.

### Phenotype-based phenome-wide association tests

We performed phenotype-based phenome-wide association analyses (Gao et al., 2023), testing for differences in a wide range of clinical diagnoses (phecodes) between groups that associate with individuals with BPD that died by suicide. Multivariable logistic regression models controlling for age, sex, and the square root-transformed total number of diagnostic codes were used to test for diagnostic differences between groups. The transformed number of total ICD codes was included as a proxy for the total number of health encounters, to account for the potential influence of informative presence bias (Goldstein, Bhavsar, Phelan, & Pencina, 2016). Differences in healthcare utilization patterns can arise for many reasons such as access to healthcare, socioeconomic status, or simply differing health needs. Some individuals in the Utah cohort are frequent users of medical services (e.g. regularly visiting doctors or managing chronic conditions). Conversely, others rarely seek medical attention, leading to more sparse EHRs. Since individuals with ‘poorer health’ tend to have more frequent healthcare visits, more data are likely recorded for them compared to other individuals, we accounted for this potential bias. Since some phecodes have a low frequency within groups, Firth's penalized maximum likelihood logistic regression was used to reduce bias (Heinze & Schemper, 2002). All analyses were conducted in R (https://www.r-project.org/).

Three different comparisons were made (SUI + BPD *v.* SUI − BPD; SUI + BPD *v.* CTRL + BPD; SUI + BPD *v.* SUI + PD − BPD). In total, 1733 phecodes were compared between SUI + BPD *v.* SUI − BPD. With Bonferroni correction for 1730 tests, *p* values below 2.89 × 10^−5^ were considered significant. In total, 1564 phecodes were compared between SUI + BPD *v.* CTRL + BPD. With Bonferroni correction for 1564 tests, *p* values below 3.2 × 10^−5^ were considered significant. In total, 1577 phecodes were compared between SUI + BPD *v.* SUI + PD − BPD. With Bonferroni correction for 1577 tests, *p* values below 3.17 × 10^−5^ were considered significant. Numbers of phecodes tested differ between comparisons based on prevalence. If the phecode was not present in either group compared it was excluded from the analysis.

We also performed within-sex, stratified analyses with multivariable logistic regression models controlling for the square root-transformed total number of diagnostic codes and age within the comparisons described above. In total, 1648 phecodes (female) and 1624 phecodes (male) were compared between SUI + BPD *v.* SUI − BPD, with Bonferroni correction for multiple testing and *p* values below 3.03 × 10^−5^ were considered significant for females and 3.08 × 10^−5^ for males. In total, 1473 phecodes (female) and 1214 phecodes (male) were compared between SUI + BPD *v.* CTRL + BPD, with Bonferroni correction for multiple testing and *p* values below 3.39 × 10^−5^ were considered significant for females and 4.12 × 10^−5^ for males. In total, 1480 phecodes (female) and 1318 phecodes (male) were compared between SUI + BPD *v.* CTRL + BPD, with Bonferroni correction for multiple testing and *p* values below 3.38 × 10^−5^ were considered significant for females and 3.79 × 10^−5^ for males.

## Results

### Sample characteristics

BPD diagnoses were present in 3.8% of suicide deaths and among 0.3% of controls. Other personality disorder diagnoses (excluding BPD) were present in 6.0% of cases and 1% of controls. On average members of the SUI + BPD group were significantly younger than the three comparison groups (adjusted *p* values <0.03), and were significantly more likely to be female (adjusted *p* values <0.0003; Table 1). The prevalence of each phecode for each group is given in online Supplementary Table S2. Age at death for each group is also now reported in Table 1. The mean time to suicide death from the first year BPD was diagnosed to the year of suicide death 5.07 years (5.30 s.d.). In total, 126 individuals (33% of suicide deaths with BPD diagnoses) died by suicide within a year of their first known diagnosis of BPD.

**Table 1.** Demographics and diagnostic characteristics of the sample

	SUI + BPD	SUI−BPD	SUI + PD−BPD	CTRL + BPD
Total #	379	9468	589	280
% Female	68.3	21.2	29.5	54.3
Mean age (s.d.)	37.6 (11.7)	43.6 (17.0)	43.2 (13.3)	39.7 (12.3)
Mean # diagnostic codes in EHR (s.d.)	140.5 (122.9)	43.3 (52.6)	87.2 (84.1)	141.2 (116.9)
% Known veteran	4.0	15.2	10.5	*
Race (%)				
White	94.5	94.2	95.1	93.6
Black	*	0.7	*	*
American Indian/Alaska Native	*	1.6	*	*
Pacific Islander	*	0.5	*	*
Asian	*	0.9	*	*
Multiple	*	0.5	*	4.6
Unknown	*	1.3	*	*
Ethnicity (%)				
Spanish/Hispanic	5.0	6.5	7.3	11.1
Education (%)				
Some high school	10.8	15.9	13.2	8.9
High school degree	33.5	38.1	38.2	27.9
Some college	39.6	28.8	27.8	23.6
College degree	8.2	8.9	9.2	6.8
Post college	5.0	6.7	9.2	*
Unknown	*	1.3	2.2	30.7
Marital status (%)				
Single	37.7	30.4	27.7	3.9
Married	25.6	37.4	31.4	3.9
Divorced	30.9	24.5	33.8	5.0
Separated	2.6	2.3	2.5	*
Widowed	*	4.6	2.9	*
Unknown	*	0.7	*	86.4
Suicide method (%)				
Firearm related	21.4	53.1	35.1	NA
Asphyxiation	22.2	23.8	24.6	NA
Overdose/poisoning	47.5	17.7	31.4	NA
Violent trauma	6.1	3.7	6.8	NA
Other	*	1.4	1.9	NA

In compliance with the Utah Resource for Genetic and Epidemiologic Research (RGE) standards, numbers smaller than 11 individuals cannot be reported and are indicated with an asterisk.

### Model comparisons

**Figure 1.**
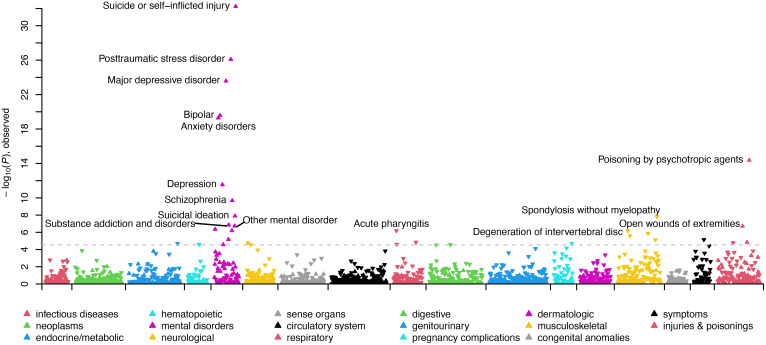
Manhattan plot comparing clinical phenotypes (Phecodes) in the electronic health record of SUI + BPD *v*. SUI − BPD. Phenotypes are grouped into 17 color-coded clinical categories along the *x*-axis. The *y*-axis represents the −log10 (*p* value) of the association between clinical category and SUI + BPD *v*. SUI − BPD. Arrows pointed upwards and indicate phenotypes that are elevated in SUI + BPD; whereas downward arrows indicate elevated phenotypes among SUI − BPD. The dashed line indicates the significance threshold with top significant clinical phenotypes in each clinical category labeled.

In the SUI + BPD *v.* SUI − BPD comparison, 17 phecodes related to psychiatric disorders (posttraumatic stress disorder [PTSD], major depressive disorder [MDD]/depression, schizophrenia/psychosis, bipolar, anxiety, agoraphobia/social phobia/panic disorder, obsessive-compulsive disorders, and dissociative disorder), substance use disorders, suicidal ideation, and previous suicide attempts were statistically significantly overrepresented in suicide deaths *with* BPD diagnoses (see Fig. 1; Table 2; online Supplementary Table S3). Conversely, 16 phecodes related to pain (acute and chronic), inflammation, and type 2 diabetes were statistically significantly overrepresented in suicide deaths *without* BPD diagnoses (see Fig. 1; online Supplementary Table S3). In the female-specific analysis, 13 phecodes related to psychiatric disorders (PTSD, MDD, anxiety, schizophrenia/psychosis, bipolar), substance use disorders, hypopotassemia, and previous suicide attempts were statistically significantly overrepresented in female suicide deaths with BPD diagnoses (online Supplementary Table S4). Three phecodes related to inflammation and premature labor were statistically significantly overrepresented in female suicide deaths without BPD diagnoses (online Supplementary Table S4). In the male-specific analysis, nine phecodes related to MDD/depression, anxiety, bipolar, suicidal ideation and previous suicide attempts, and insomnia were statistically significantly overrepresented in male suicide deaths with BPD diagnoses and no phecodes significantly differed in male suicide deaths without BPD diagnoses (online Supplementary Table S5).

**Table 2.** Phecode information, means and significant model results for each group comparison

Phecode name	Mean group 1 (s.d.)	Mean group 2 (s.d.)	Phecode	Phecode category	*p* Value	OR (LCI–UCI)
SUI + BPD *v*. SUI – BPD	SUI + BPD	SUI – BPD			<2.89 × 10^−5^	
Suicide or self-inflicted injury	0.82 (0.39)	0.2 (0.40)	297.2	Mental disorders	5.76 × 10^−33^	5.15 (3.87–6.93)
Posttraumatic stress disorder	0.5 (0.50)	0.05 (0.23)	300.9	Mental disorders	8.18 × 10^−27^	4.34 (3.34–5.62)
Major depressive disorder	0.86 (0.35)	0.28 (0.45)	296.22	Mental disorders	2.64 × 10^−24^	4.57 (3.34–6.36)
Bipolar	0.59 (0.49)	0.12 (0.32)	296.1	Mental disorders	2.63 × 10^−20^	3.21 (2.51–4.11)
Anxiety disorders	0.46 (0.50)	0.08 (0.28)	300	Mental disorders	5.08 × 10^−20^	3.25 (2.54–4.15)
Poisoning by psychotropic agents	0.65 (0.48)	0.14 (0.35)	969	Injuries and poisonings	4.22 × 10^−15^	2.77 (2.15–3.58)
Depression	0.91 (0.29)	0.39 (0.49)	296.2	Mental disorders	2.95 × 10^−12^	3.54 (2.42–5.32)
Schizophrenia	0.23 (0.42)	0.04 (0.20)	295.1	Mental disorders	2.07 × 10^−10^	2.83 (2.08–3.88)
Suicidal ideation	0.6 (0.49)	0.16 (0.37)	297.1	Mental disorders	1.26 × 10^−8^	2.07 (1.61–2.67)
Spondylosis without myelopathy	0.14 (0.35)	0.1 (0.31)	721.1	Musculoskeletal	1.47 × 10^−8^	0.36 (0.25–0.52)
Other mental disorder	0.52 (0.50)	0.11 (0.32)	306	Mental disorders	1.40 × 10^−7^	2.04 (1.57–2.66)
Substance addiction and disorders	0.73 (0.45)	0.26 (0.44)	316	Mental disorders	1.84 × 10^−7^	2.01 (1.54–2.64)
Open wounds of extremities	0.42 (0.49)	0.14 (0.35)	871	Injuries and poisonings	1.91 × 10^−7^	1.94 (1.52–2.48)
Agoraphobia, social phobia, and panic disorder	0.42 (0.49)	0.08 (0.27)	300.12	Mental disorders	4.45 × 10^−7^	1.98 (1.53–2.57)
Psychosis	0.32 (0.47)	0.07 (0.25)	295.3	Mental disorders	6.34 × 10^−7^	2.06 (1.56–2.70)
Degeneration of intervertebral disc	0.2 (0.40)	0.12 (0.33)	722.6	Musculoskeletal	6.40 × 10^−7^	0.45 (0.32–0.62)
Acute pharyngitis	0.31 (0.46)	0.15 (0.36)	465.2	Respiratory	6.59 × 10^−7^	0.51 (0.37–0.67)
Pain in joint	0.51 (0.50)	0.27 (0.45)	745	Musculoskeletal	1.36 × 10^−6^	0.51 (0.39–0.67)
Displacement of intervertebral disc	0.13 (0.34)	0.1 (0.30)	722.1	Musculoskeletal	2.49 × 10^−6^	0.44 (0.31–0.63)
Obsessive-compulsive disorders	0.16 (0.36)	0.02 (0.15)	300.3	Mental disorders	6.75 × 10^−6^	2.43 (1.67–3.50)
Neuralgia, neuritis, and radiculitis NOS	0.04 (0.20)	0.03 (0.16)	766	Symptoms	7.04 × 10^−6^	0.28 (0.15–0.50)
Spinal stenosis	0.03 (0.18)	0.03 (0.17)	720	Musculoskeletal	7.53 × 10^−6^	0.26 (0.13–0.48)
Postnasal drip	0	0.004 (0.06)	475.9	Respiratory	1.42 × 10^−5^	0.01 (0.00–0.14)
Poisoning by anticonvulsants and anti-Parkinsonism drugs	0.24 (0.43)	0.03 (0.17)	966	Injuries and poisonings	1.44 × 10^−5^	2.11 (1.51–2.92)
Fracture of unspecified bones	0.11 (0.31)	0.06 (0.24)	809	Injuries and poisonings	1.61 × 10^−5^	0.42 (0.28–0.63)
Acute pain	0.21 (0.41)	0.07 (0.25)	338.1	Neurological	1.69 × 10^−5^	0.47 (0.33–0.67)
Threatened premature labor	0.04 (0.21)	0.01 (0.10)	636.1	Pregnancy complications	2.00 × 10^−5^	0.28 (0.14–0.52)
Type 2 diabetes with neurological manifestations	0 (0.05)	0.01 (0.12)	250.24	Endocrine/metabolic	2.00 × 10^−5^	0.05 (0.00–0.27)
Acute sinusitis	0.2 (0.40)	0.09 (0.29)	464	Respiratory	2.50 × 10^−5^	0.52 (0.38–0.71)
Lymphadenitis	0.08 (0.27)	0.04 (0.20)	289.4	Hematopoietic	2.53 × 10^−5^	0.40 (0.25–0.63)
Dissociative disorder	0.07 (0.26)	0 (0.06)	303.1	Mental disorders	2.68 × 10^−5^	3.91 (2.09–7.27)
Diverticulosis	0.04 (0.20)	0.08 (0.27)	562.1	Digestive	2.77 × 10^−5^	0.33 (0.18–0.57)
Chronic pain	0.37 (0.48)	0.13 (0.34)	338.2	Neurological	2.84 × 10^−5^	0.52 (0.38–0.71)
SUI + BPD *v*. CTRL + BPD	SUI + BPD	CTRL + BPD			<3.2 × 10^−5^	
Suicide or self-inflicted injury	0.82 (0.39)	0.56 (0.50)	297.2	Mental disorders	9.75 × 10^−13^	3.73 (2.58–5.44)
Poisoning by psychotropic agents	0.65 (0.48)	0.45 (0.50)	969	Injuries and poisonings	1.39 × 10^−7^	2.55 (1.79–3.64)
Hypopotassemia	0.43 (0.5)	0.28 (0.45)	276.14	Endocrine/metabolic	8.10 × 10^−6^	2.36 (1.61–3.49)
Infectious and parasitic complications affecting pregnancy	0.01 (0.10)	0.06 (0.24)	647	Pregnancy complications	1.33 × 10^−5^	0.120 (0.04–0.33)
Poisoning by anticonvulsants and anti-Parkinsonism drugs	0.24 (0.43)	0.13 (0.34)	966	Injuries and poisonings	2.03 × 10^−5^	2.65 (1.68–4.27)
SUI + BPD *v*. SUI – BPD + PD	SUI + BPD	SUI − BPD + PD			<3.17 × 10^−5^	
Posttraumatic stress disorder	0.50	0.42	300.9	Mental disorders	8.48 × 10^−9^	2.77 (1.96–3.93)

**Figure 2.**
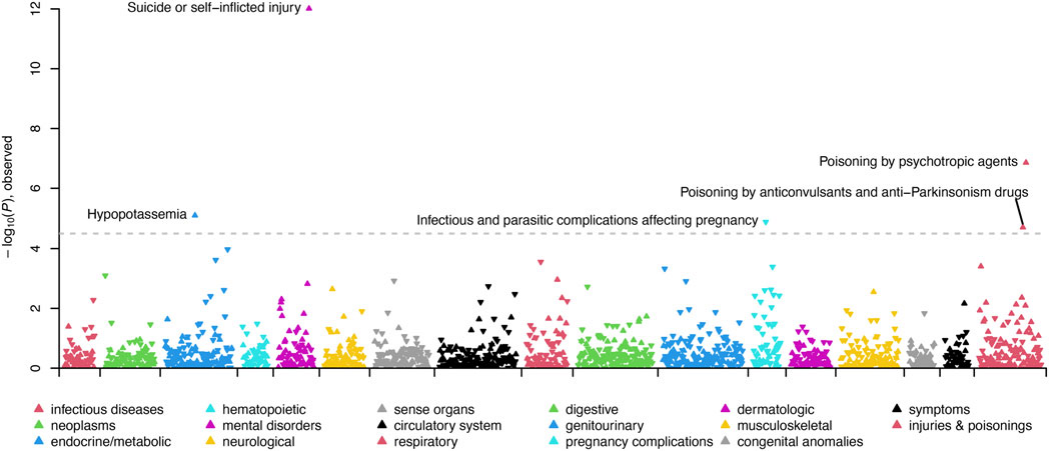
Manhattan plot comparing clinical phenotypes (Phecodes) in the electronic health record of SUI + BPD *v*. CTRL + BPD. Phenotypes are grouped into 17 color-coded clinical categories along the *x*-axis. The *y*-axis represents the −log10 (*p* value) of the association between clinical category and SUI + BPD *v*. CTRL + BPD. Arrows pointed upwards and indicate phenotypes that are elevated in SUI + BPD; whereas downward arrows indicate elevated phenotypes among CTRL + BPD. The dashed line indicates the significance threshold with top significant clinical phenotypes in each clinical category labeled.

In the SUI + BPD *v.* CTRL + BPD comparison, four phecodes were statistically significantly overrepresented in suicide deaths with BPD diagnoses related to previous suicide attempts (including overdoses) or self-injury and hypopotassemia (see Fig. 2; Table 2; online Supplementary Table S6). One phecode was more prevalent in controls with BPD diagnoses ‘infectious and parasitic complications affecting pregnancy’. In the female-specific analyses, the same phecodes as above were significant, with the addition of substance use disorders in the female suicide deaths with BPD diagnoses (online Supplementary Table S7). No phecodes were significantly different in the male-specific analyses (online Supplementary Table S8).

**Figure 3.**
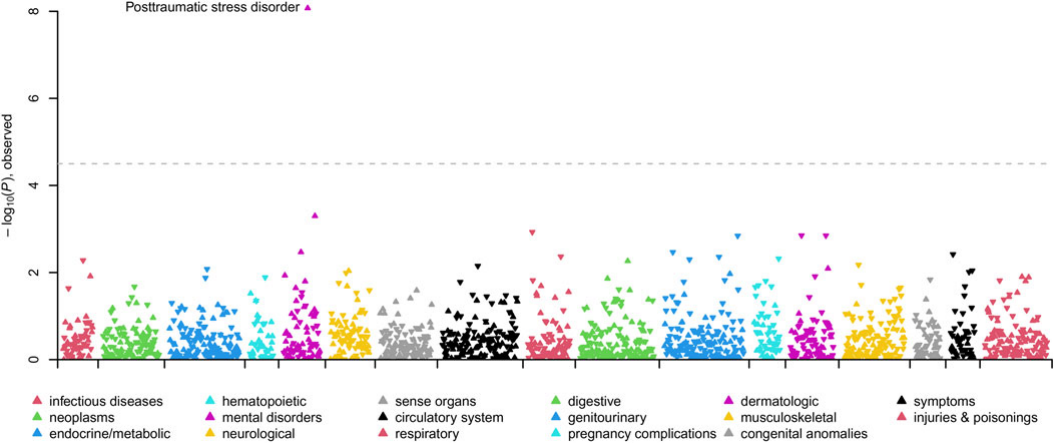
Manhattan plot comparing clinical phenotypes (Phecodes) in the electronic health record of SUI + BPD *v*. SUI + PD − BPD. Phenotypes are grouped into 17 color-coded clinical categories along the *x*-axis. The *y*-axis represents the −log10 (*p* value) of the association between clinical category and SUI + BPD *v*. SUI + PD − BPD. Arrows pointed upwards and indicate phenotypes that are elevated in SUI + BPD; whereas downward arrows indicate elevated phenotypes among SUI + PD − BPD. The dashed line indicates the significance threshold with top significant clinical phenotypes in each clinical category labeled.

In the SUI + BPD *v.* SUI + PD − BPD comparison, one phecode was statistically significantly overrepresented in suicide deaths with BPD diagnoses, ‘PTSD’. No phecodes were statistically significantly overrepresented in suicide deaths with other personality disorder diagnoses (Fig. 3; Table 2; online Supplementary Table S9). In the female-specific analysis, the PTSD phecode remained statistically significantly overrepresented in suicide deaths with BPD diagnoses (online Supplementary Table S10) and no phecodes were significant in the male-specific analysis (online Supplementary Table S11).

## Discussion

The present study used a unique, population-based suicide data resource to examine diagnostic correlates among persons with a BPD diagnosis who died by suicide. We compared this high-risk group to a number of clinically meaningful comparison groups in an attempt to better understand what characterizes risk among members of this particularly vulnerable clinical population. Even using a conservative approach to correct for multiple comparisons, differences emerged between persons with BPD who died by suicide and all other groups examined.

Those in the SUI + BPD group appeared more likely to present with co-occurring psychiatric diagnoses, and have a documented history of self-harm in the medical system prior to death, relative to those in the SUI – BPD group. Self-harm phecodes included ‘Open wounds of extremities (871)’, ‘Suicide or self-inflicted injury (297.2)’, and ‘Poisoning by psychotropic agents (969)’; these encompass a broad range of self-harm behaviors which included both non-suicidal and suicidal intent prior to the suicide death event.

These results align with an extensive literature documenting prevalent co-occurrence of BPD and self-injury, as well as high comorbidity of BPD with other psychiatric conditions like substance use. For example, studies suggest that as many as 70–95% of persons with BPD engage in some form of self-harm (whether non-suicidal or suicidal in nature; Goodman et al., 2017; Oumaya et al., 2008), and approximately half of persons with BPD also meet criteria for current alcohol use disorder (Trull, Sher, Minks-Brown, Durbin, & Burr, 2000; Trull et al., 2018). These patterns are noteworthy for two reasons. First, substance misuse and self-harm each appear to elevate risk for suicide in their own right (Grandclerc, De Labrouhe, Spodenkiewicz, Lachal, & Moro, 2016; Rizk, Herzog, Dugad, & Stanley, 2021), and, when combined with BPD, may elevate risk still further. Second, substance misuse and self-injury often each serve *as means for dampening or avoiding negative affect* (Hooley & Franklin, 2018; Weiss et al., 2022). Emotion dysregulation is arguably the most central feature of BPD (Linehan, 1993) and our results demonstrate that BPD is associated with conditions marked by emotional and psychological distress at an elevated rate (even in comparison to other persons who die by suicide).

Interestingly, persons who died by suicide *without* BPD appear to have more frequently documented medical comorbidity and phecodes related to pain. There is an extensive literature documenting chronic pain as a risk factor for suicidal ideation, attempts, and suicide deaths (Kohrt, Griffith, & Patel, 2018), and recent evidence indicates that the risk of suicide increases twofold among individuals with chronic pain (Chincholkar & Blackshaw, 2023). Recent latent class analyses have found that physical pain and medical conditions constitute a substantial group of suicide deaths (Xiao et al., 2024). It is therefore noteworthy that individuals in the BPD + suicide group were relatively *less* likely to experience such conditions; particularly as individuals with BPD are overrepresented among those seeking medical care (see e.g. Sansone & Sansone, 2015). Future research should examine whether the pathways to suicide may be more closely tied to emotional distress among persons with BPD, as compared to physical illness and/or pain among those who die by suicide without BPD.

Results comparing the SUI + BPD to the CTRL + BPD group indicate that history of previous suicide attempts and/or self-injury was significantly more common among those who died by suicide. This aligns with extant research indicating that more frequent engagement with self-injurious behaviors (suicidal and non-suicidal) advances risk for death by suicide more robustly than other known predictors and correlates (Franklin et al., 2017; Guan, Fox, & Prinstein, 2012). It may be that persons with BPD who go on to die by suicide are more likely to engage in, or perhaps persistent in their self-injury, relative to living persons with a BPD history. Those in the SUI + BPD group also had higher incidents of hypopotassemia, whereas those in the CTRL + BPD were more likely to have medical complications affecting a pregnancy. It may be that persons in the SUI + BPD group were more likely to be hospitalized or treated in emergency departments where electrolytes are often assessed via blood test. There may also have been differences in medications prescribed to members of each of these groups such that potassium may have been more impacted among those with SUI + BPD. This may be an indirect signal of differences in treatment patterns for these groups. Diuretic use and patterns of binging/purging may also result in low potassium, and may have contributed to differences across groups.

Finally, when comparing the SUI + BPD group to those who died by suicide with another PD diagnosis, differences only emerged with respect to the PTSD phecode. Studies suggest that approximately 30% of those with BPD have comorbid PTSD (Pagura et al., 2010). A review by Frías and Palma (2015) suggests that rates of PTSD are not generally elevated among persons with BPD as compared to other personality disorder diagnoses. However, results from our sample indicate that PTSD was more common among persons with BPD *who die by suicide* as compared with both the other PD group and the SUI–BPD group. Future research should examine whether trauma exposure and PTSD symptoms interact with other suicide risk factors in the context of BPD to elevate risk of death. It is possible that the presence of BPD may compound symptoms of trauma so as to maximize their negative impact.

Our work is complementary to recent research by Xiao et al. (2024) which identified five latent classes in suicide decedents and provides further support for a multifaceted approach to suicide prevention. Here, we comprehensively assessed diagnosed health conditions among suicide decedents who had interactions with the healthcare system. For example, the Utah medical examiner reports (data similar to those included in the NVDRS/Xiao et al. study) indicate BPD among only eight individuals. However, the additional and complimentary diagnostic data gleaned from electronic heath records indicate that 379 individuals who died by suicide had a documented history of BPD diagnoses. It is possible that these individuals represent an additional subtype within class 1 ‘mental health and substance problems’ or class 2 ‘mental health problems’ identified by Xiao et al.; however, additional research is needed to investigate this hypothesis.

There are a number of study limitations that warrant discussion. First, although medical charts can and do provide essential and valid information, documented mental health diagnoses may not have been assigned by a mental health professional or following a rigorous diagnostic assessment. Emergency departments and medical providers are not typically trained on how to identify BPD or provide appropriate referrals (Cases, Lafont Rapnouil, Gallini, Arbus, & Salles, 2020). Recent work has also highlighted how stigma surrounding BPD can lead to medical neglect and misdiagnosis (see e.g. Masland et al., 2023). Thus, there are likely many persons in the USMRS who may have met criteria for BPD or another psychiatric disorder yet were undiagnosed, and many persons who were incorrectly diagnosed (reflected in the lower prevalence rates of BPD and other PDs in the USMRS relative to the general population; Lenzenweger, Lane, Loranger & Kessler, 2007). This may be particularly true for males with respect to BPD. Though prevalence rates of BPD in men and women are roughly equal (Lenzenweger et al., 2007; Torgersen, Kringlen, & Cramer, 2001), approximately 75% of persons receiving the diagnosis are women (APA, 2022), indicating potential bias in sampling and diagnosis (Masland et al., 2023). (Also of note, our BPD + suicide group is 32% male [*n* = 120]. This sample size may be insufficient to provide adequate statistical power for detecting associations with clinical diagnoses. With such a small cohort, we are likely underpowered to detect true associations that actually exist in the broader population.) ICD codes may also not be applied with specificity for billing in comparison to a comprehensive diagnostic interview – particularly when disentangling closely related psychopathology such as distinguishing PDs. Relatedly, many individuals may not have disclosed a history of self-inflicted injury to a medical provider nor are these behaviors universally screened for in all health care settings, and thus, such history may have gone undocumented among some percentage of our sample. Both of these phenomena would result in bias toward the null due to the inability to include true BPD cases and/or correctly document self-inflicted injury.

Relying on EHR data may also have resulted in incomplete coverage with respect to diagnostic data among older participants in the sample, as electronic records came in to use for the USRMS in 1996. The mean age for our identified groups is somewhat young, and thus, we likely capture EHR data across much of the adult life span of most of our participants. Nevertheless, results may be most relevant to younger cohorts.

Another limitation is that we did not investigate the timing of phecode documentation relative to participant death except for first diagnosis of BPD relative to death by suicide. Future research should examine baseline and time-varying indicators of risk *longitudinally* so as to gain a better understanding of both immediate and cumulative risk, as well as onset, persistence, and offset of medical and mental health diagnoses relative to suicide death. Generally, relying on medical records as the only data source also limits the amount of contextual information we can glean. Future research should combine this type of archival information with that obtained via other data collection methods (e.g. bloodspot data to assess genetic sources of risk available through the USMRS; prospective data acquired from family members). Data regarding marital status were less complete for control individuals in our sample, which may have contributed to observed differences in marriage rates between groups. Thus, this finding should be interpreted with caution. Finally, we would like to note limitations related to the generalizability of our findings. Although the USMRS provides an unparalleled resource, racial and ethnic diversity is limited in Utah (U.S. Census data, 2023) and our data reflect that. The patterns we observed may not generalize readily to non-White groups, which is especially unfortunate, as the impacts of stigma, discrimination, and marginalization impact the manifestation, assessment, conceptualization, and treatment of both psychopathology and medical illness (see e.g. Chen & Mallory, 2021).

Our findings indicate that history of engagement with self-injury, comorbidity with other psychiatric conditions, and engagement with maladaptive emotion regulation strategies may be particularly frequent among persons with BPD who die by suicide – even relative to other persons who die by suicide. Although our study was purely correlational in nature and we cannot speak to causality, results of the present study highlight several areas for further inquiry. Future directions for this work include examining patterns of health service contacts, more thoroughly characterizing patterns of comorbidity (e.g. examining the frequencies of specific combinations of comorbid diagnoses represented across groups), investigating patters of medication usage, and examining age-period-cohort effects with longitudinal modeling to understand changes over time.

Our data highlight that chronically suicidal people with BPD kill themselves at an elevated rate. Prevention efforts aimed at addressing self-harm by providing adaptive coping strategies may aid in reducing the burden associated with BPD, and potentially reduce risk for suicide among this population; whereas targeting physical pain and chronic medical conditions may be helpful among persons at elevated risk of suicide without BPD. Allocation resources to those with multiple co-occurring diagnoses (particularly PTSD) may be especially impactful. This work, in agreement with much previous research, elucidates the high degree of vulnerability among individuals with BPD and chronic self-harm, and the urgent need for increased understanding of the clinical and demographic nuances enhancing their risk. Continued work in this area will assist in improvements in risk detection and personalized treatments.

## Supporting information

Kaufman et al. supplementary material Kaufman et al. supplementary material

## Supplementary material

The supplementary material for this article can be found at https://doi.org/10.1017/S0033291724002034.

## References

[ref1] American Psychiatric Association (2022). Diagnostic and statistical manual of mental disorders (5th ed.). Washington, DC: American Psychiatric Association Publishing. 10.1176/appi.books.9780890425787

[ref2] Bastarache, L. (2021). Using phecodes for research with the electronic health record: From PheWAS to PheRS. Annual Review of Biomedical Data Science, 4, 1–19. 10.1146/annurev-biodatasci-122320-112352 PMC9307256 34465180

[ref3] Carroll, R. J., Bastarache, L., & Denny, J. C. (2014). R PheWAS: Data analysis and plotting tools for phenome-wide association studies in the R environment. Bioinformatics, 30(16), 2375–2376. 10.1093/bioinformatics/btu197 24733291 PMC4133579

[ref4] Cases, C., Lafont Rapnouil, S., Gallini, A., Arbus, C., & Salles, J. (2020). Evidence of practice gaps in emergency psychiatric care for borderline personality disorder: How can this be explained?. BMC Psychiatry, 20(1), 476. 10.1186/s12888-020-02892-7 32993589 PMC7526189

[ref5] Centers for Disease Control and Prevention (2023, August 10). Suicide data and statistics. Retrieved November 27, 2023, from https://www.cdc.gov/suicide/suicide-data-statistics.html

[ref6] Chen, S., & Mallory, A. B. (2021). The effect of racial discrimination on mental and physical health: A propensity score weighting approach. Social Science & Medicine *(1982)*, 285, 114308. 10.1016/j.socscimed.2021.114308 34399293 PMC8451383

[ref7] Chincholkar, M., & Blackshaw, S. (2023). Suicidality in chronic pain: Assessment and management. BJA Education, 23(8), 320–326. 10.1016/j.bjae.2023.05.005 37465233 PMC10350556

[ref8] Denny, J. C., Bastarache, L., Ritchie, M. D., Carroll, R. J., Zink, R., Mosley, J. D., … Roden, D. M. (2013). Systematic comparison of phenome-wide association study of electronic medical record data and genome-wide association study data. Nature Biotechnology, 31(12), 1102–1110. 10.1038/nbt.2749 PMC3969265 24270849

[ref9] Derbidge, C. M., & Beauchaine, T. P. (2014). A developmental model of self-inflicted injury, borderline personality, and suicide risk. In Lewis, M., & Rudolph, K. (Eds.), Handbook of developmental psychopathology (3rd edn, pp. 521–542). Boston, MA: Springer. 10.1007/978-1-4614-9608-3_26

[ref10] Franklin, J. C., Ribeiro, J. D., Fox, K. R., Bentley, K. H., Kleiman, E. M., Huang, X., … Nock, M. K. (2017). Risk factors for suicidal thoughts and behaviors: A meta-analysis of 50 years of research. Psychological Bulletin, 143(2), 187–232. 10.1037/bul0000084 27841450

[ref11] Frías, Á., & Palma, C. (2015). Comorbidity between post-traumatic stress disorder and borderline personality disorder: A review. Psychopathology, 48(1), 1–10. 10.1159/000363145 25227722

[ref12] Gao, Y. N., Coombes, B., Ryu, E., Pazdernik, V., Jenkins, G., Pendegraft, R., … Olfson, M. (2023). Phenotypic distinctions in depression and anxiety: A comparative analysis of comorbid and isolated cases. Psychological Medicine, 53(16), 7766–7774. 10.1017/S0033291723001745 37403468 PMC11251006

[ref13] Goldstein, B. A., Bhavsar, N. A., Phelan, M., & Pencina, M. J. (2016). Controlling for informed presence bias due to the number of health encounters in an electronic health record. American Journal of Epidemiology, 184(11), 847–855. 10.1093/aje/kww112 27852603 PMC5152663

[ref14] Goodman, M., Tomas, I. A., Temes, C. M., Fitzmaurice, G. M., Aguirre, B. A., & Zanarini, M. C. (2017). Suicide attempts and self-injurious behaviours in adolescent and adult patients with borderline personality disorder. Personality and Mental Health, 11(3), 157–163. 10.1002/pmh.1375 28544496 PMC5571736

[ref15] Grandclerc, S., De Labrouhe, D., Spodenkiewicz, M., Lachal, J., & Moro, M. R. (2016). Relations between nonsuicidal self-injury and suicidal behavior in adolescence: A systematic review. PLoS ONE, 11(4), e0153760. 10.1371/journal.pone.0153760 27089157 PMC4835048

[ref16] Guan, K., Fox, K. R., & Prinstein, M. J. (2012). Nonsuicidal self-injury as a time-invariant predictor of adolescent suicide ideation and attempts in a diverse community sample. Journal of Consulting and Clinical Psychology, 80(5), 842–849. 10.1037/a0029429 22845782 PMC3458144

[ref17] Heinze, G., & Schemper, M. (2002). A solution to the problem of separation in logistic regression. Statistics in Medicine. 21, 2409–2419. 10.1002/sim.1047 12210625

[ref18] Hooley, J. M., & Franklin, J. C. (2018). Why do people hurt themselves? A new conceptual model of nonsuicidal self-injury. Clinical Psychological Science, 6(3), 428–451. 10.1177/2167702617745641

[ref19] Kaufman, E. A., Crowell, S. E., & Lenzenweger, M. (2017). The development of borderline personality disorder and self-inflicted injury. In Beauchaine, T. P., & Hinshaw, S. P. (Eds.), Child and adolescent psychopathology (3rd ed., pp. 642–679). Hoboken, NJ: Wiley.

[ref20] Kohrt, B. A., Griffith, J. L., & Patel, V. (2018). Chronic pain and mental health: Integrated solutions for global problems. Pain, 159(Suppl. 1), S85–S90. 10.1097/j.pain.0000000000001296 30113952 PMC6130207

[ref21] Lenzenweger, M. F., Lane, M. C., Loranger, A. W., & Kessler, R. C. (2007). DSM-IV personality disorders in the national comorbidity survey replication. Biological Psychiatry, 62(6), 553–564. 10.1016/j.biopsych.2006.09.019 17217923 PMC2044500

[ref22] Linehan, M. M. (1993). Cognitive-behavioral treatment of borderline personality disorder. Guilford Press.

[ref23] Masland, S. R., Victor, S. E., Peters, J. R., Fitzpatrick, S., Dixon-Gordon, K. L., Bettis, A. H., … Rizvi, S. L. (2023). Destigmatizing borderline personality disorder: A call to action for psychological science. Perspectives on Psychological Science, 18(2), 445–460. https://doi-org.ezproxy.lib.utah.edu/10.1177/17456916221100464 36054911 10.1177/17456916221100464

[ref24] Oumaya, M., Friedman, S., Pham, A., Abou Abdallah, T., Guelfi, J. D., & Rouillon, F. (2008). Personnalité borderline, automutilations et suicide: Revue de la littérature [Borderline personality disorder, self-mutilation and suicide: Literature review]. L'Encephale, 34(5), 452–458. 10.1016/j.encep.2007.10.007 19068333

[ref25] Pagura, J., Stein, M. B., Bolton, J. M., Cox, B. J., Grant, B., & Sareen, J. (2010). Comorbidity of borderline personality disorder and posttraumatic stress disorder in the U.S. population. Journal of Psychiatric Research, 44(16), 1190–1198. 10.1016/j.jpsychires.2010.04.016 20537660 PMC4209725

[ref26] Paris J. (2019). Suicidality in borderline personality disorder. Medicina, 55(6), 223. 10.3390/medicina55060223 31142033 PMC6632023

[ref27] Pascual, J. C., Córcoles, D., Castaño, J., Ginés, J. M., Gurrea, A., Martín-Santos, R., & Bulbena, A. (2007). Hospitalization and pharmacotherapy for borderline personality disorder in a psychiatric emergency service. Psychiatric Services, 58(9), 1199–1204. 17766566 10.1176/ps.2007.58.9.1199

[ref28] Rizk, M. M., Herzog, S., Dugad, S., & Stanley, B. (2021). Suicide risk and addiction: The impact of alcohol and opioid use disorders. Current Addiction Reports, 8(2), 194–207. 10.1007/s40429-021-00361-z 33747710 PMC7955902

[ref29] Sansone, R. A., & Sansone, L. A. (2015). Borderline personality disorder in the medical setting: Suggestive behaviors, syndromes, and diagnoses. Innovations in Clinical Neuroscience, 12(7–8), 39–44. 26351624 PMC4558791

[ref30] Sansone, R. A., Farukhi, S., & Wiederman, M. W. (2011). Utilization of primary care physicians in borderline personality. General Hospital Psychiatry, 33(4), 343–346. 10.1016/j.genhosppsych.2011.04.006 21762830

[ref31] Torgersen, S., Kringlen, E., & Cramer, V. (2001). The prevalence of personality disorders in a community sample. Archives of General Psychiatry, 58(6), 590–596. 10.1001/archpsyc.58.6.590 11386989

[ref32] Trull, T. J., Sher, K. J., Minks-Brown, C., Durbin, J., & Burr, R. (2000). Borderline personality disorder and substance use disorders: A review and integration. Clinical Psychology Review, 20, 235–253. 10.1016/S0272-7358(99)00028-8 10721499

[ref33] Trull, T. J., Freeman, L. K., Vebares, T. J., Choate, A. M., Helle, A. C., & Wycoff, A. M. (2018). Borderline personality disorder and substance use disorders: An updated review. Borderline Personality Disorder and Emotion Dysregulation, 5, 15–26. 10.1186/s40479-018-0093-9. 30250740 PMC6145127

[ref34] US Census Data (2023). QuickFacts Utah. Retrieved March 21, 2024, from: https://www.census.gov/quickfacts/fact/table/UT/PST045223

[ref35] Utah Department of Health and Human Services, Indicator-Based Information System (IBIS) for Public Health (2023, January 24). Retrieved November 27, 2023, from: http://ibis.health.utah.gov

[ref36] Wedig, M., Silverman, M., Frankenburg, F., Reich, D., Fitzmaurice, G., & Zanarini, M. (2012). Predictors of suicide attempts in patients with borderline personality disorder over 16 years of prospective follow-up. Psychological Medicine, 42(11), 2395–2404. doi: 10.1017/S0033291712000517 22436619 PMC3600404

[ref37] Weiss, N. H., Kiefer, R., Goncharenko, S., Raudales, A. M., Forkus, S. R., Schick, M. R., & Contractor, A. A. (2022). Emotion regulation and substance use: A meta-analysis. Drug and Alcohol Dependence, 230, 109131. 10.1016/j.drugalcdep.2021.109131 34864568 PMC8714680

[ref38] Xiao, Y., Bi, K., Yip, P. S. F., Cerel, J., Brown, T. T., Peng, Y., … Mann, J. J. (2024). Decoding suicide decedent profiles and signs of suicidal intent using latent class analysis. JAMA Psychiatry, 81(6), 595–605. 38506817 10.1001/jamapsychiatry.2024.0171 PMC10955339

